# Water Physiological Characteristics and Tonoplast Transporter Expression Profiles During Granulation in ‘Newhall’ Navel Oranges

**DOI:** 10.3390/foods15213848

**Published:** 2026-10-28

**Authors:** Xiong Lin, Qingjiang Wei, Wenjia Xie, Yingying Chen, Jiansheng Shen, Ming Chen, Jinyin Chen, Qiaoli Ma

**Affiliations:** 1 Jiangxi Provincial Key Laboratory for Postharvest Storage and Preservation of Fruits & Vegetables, Collaborative Innovation Center of Postharvest Key Technology and Quality Safety of Fruits and Vegetables, Jiangxi Agricultural University, Nanchang 330045, China; 2 Jinhua Laboratory of Biological Seed Industry and Modern Agricultural Machinery, Jinhua Academy of Agricultural Sciences, Jinhua 321000, China

**Keywords:** citrus granulation, water potential, vacuolar membrane transporter, osmolyte, water transport

## Abstract

Granulation with drying juice sacs is a common and seriously quality-affecting physiological disorder in citrus fruit. However, the intrinsic connection between it and the physiological regulation of fruit water, sugars and acids is still poorly understood. This study systematically quantified dynamic changes in key water status and sugar-acid transport parameters across granulation development to uncover how imbalanced water and sugar-acid metabolism and disrupted cellular hydration trigger granulation. In this study, ‘Newhall’ navel oranges (*Citrus sinensis* Osbeck) were used as the experimental material. The dynamic changes of key water and sugar/acid transport physiological indicators during the granulation process were systematically determined, aiming to clarify the physiological mechanism of water and sugar/acid metabolism imbalance and cell hydration state disorder during the occurrence of granulation. Both granulation rate and index increased significantly over time, reaching 84.98% and 18.33 by day 200, respectively. Granulated fruits exhibited accelerated water loss, a marked decrease in pulp water potential, and a progressively widening water potential gradient between peel and pulp. Physiological analysis revealed a significant decline in organic osmolytes (sugars, organic acids, and soluble proteins) in both tissues, alongside a redistribution of mineral elements (K, Ca, Mg) from peel to pulp during granulation. Gene expression profiling showed that granulation progression was associated with tissue-specific upregulation of several tonoplast intrinsic protein (*CsTIP*) genes (e.g., *CsTIP1;1*, *CsTIP1;2*, *CsTIP2;1*) and specific vacuolar membrane transporters for sugars and acids. Correlation analysis indicated strong relationships among water status, osmolyte dynamics, and vacuolar transporter gene expression. These findings demonstrate that the expanding peel–pulp water potential gradient drives osmotic disturbance and alters vacuolar transport activity, which collectively induce juice sac desiccation and granulation. These insights provide a physiological and molecular basis for developing water-management strategies to mitigate this quality disorder in citrus.

## 1. Introduction

Citrus fruits, the most consumed fruit worldwide, are prone to a physiological disorder known as juice sac granulation (also referred to as segment drying or puffing sometimes) during postharvest storage [1,2]. Affected fruits show no obvious external symptoms; internally, however, the juice sacs exhibit thickened cell walls, reduced firmness, faded coloration, and shriveling due to moisture loss [3]. Consequently, the juice yield decreases markedly and the sugar-acid flavor becomes insipid [4]. These deteriorations severely impair the nutritional quality, edibility, and commercial value of the fruit. Considerable research has been conducted on postharvest granulation in various citrus cultivars. These studies have predominantly explored the disorder in terms of its phenotype, occurrence, and mechanisms [5], with particular emphasis on tissue structure, cell morphology [6], cell wall material accumulation [7,8], sugar-acid metabolism [9], and senescence regulation [3,10]. However, little attention has been given to the water physiological changes, especially the correlation between the differential water physiology of the peel and pulp and the development of granulation.

Water transport from the pulp to the peel during storage is considered one of the important factors contributing to granulation. During postharvest senescence of citrus fruits, the initial deterioration occurs in the pulp tissue, manifested as dehydration and shrinkage of juice sacs and alterations in internal constituents. This is attributed to the substantial translocation of water and sugar-acid from the pulp to the peel through the vascular tissues (including the columella and albedo), which sustains peel physiological activity but imposes water stress on the pulp, leading to physiological disorders [11,12]. Using tritiated water (^3^H_2_O) as a tracer, previous studies simulated water movement among pedicel, peel, and pulp (juice sacs) of ‘Shatian’ pummelo during storage. Bidirectional water exchange occurred between segments, and between the pulp and the peel, but the pulp-to-peel transfer rate was higher in granulated fruit than in normal fruit [13]. Recent research has analyzed water physiological changes in different citrus types (including loose-skinned and tight-skinned varieties) during storage [14]. Significant alterations were observed in inter-tissue water potential, osmotic regulators such as sugars, organic acids, soluble proteins, amino acids, and inorganic ions, as well as in the expression patterns of aquaporin genes regulating water transport [15,16]. These findings provide important clues for understanding the water physiological changes and material transport associated with postharvest granulation in citrus fruit.

The central vacuole is the largest subcellular organelle in mature plant cells, occupying up to 90% of the cell volume [17]. It stores large amounts of water and various solutes—including sugars, organic acids, amino acids, and pigments—and thus plays a crucial role in maintaining osmotic regulation, cell morphology and determining the quality traits of horticultural products [18,19]. The tonoplast harbors a large number of transporters, including aquaporins, sugar transporters, and acid transporters, which are specifically responsible for the transmembrane transport of various solutes [20,21]. Tonoplast intrinsic proteins (TIPs) are the most abundant proteins on the tonoplast, accounting for 30–50% of the total tonoplast protein and mediating water transport across the membrane. Plant genomes encode multiple *TIP* genes, ranging from 6 to 35 per species. Based on amino acid sequence homology and substrate specificity, TIPs in higher plants are divided into five subfamilies [22]. The sweet orange genome (http://citrus.hzau.edu.cn/index.php, accessed on 15 January 2021.) encodes eight *TIP* homologs, including three *TIP1* (*TIP1;1*, *TIP1;2*, and *TIP1;3*), two *TIP2* (*TIP2;1* and *TIP2;2*), and one each of *TIP3*, *TIP4*, and *TIP5*. Some vacuolar sugar and acid transporters identified in citrus fruit—such as *ABGC15*, *ABCC4*, *Dict2.1*, *ALMT4*, and *CLCc*—have been shown to participate in vacuolar citrate accumulation. Others, including *NPF5.1*, *NPF8.3*, *SUT4*, *STP7*, *TMT2*, and *ERD6Ls*, are involved in the vacuolar compartmentation of soluble sugars. Collectively, these transporters regulate the flux of citrate, glucose, and sucrose across the tonoplast [19,23,24]. The expression and activity of these transporters directly determine vacuolar solute composition, thereby modulating cellular osmotic potential and water balance. However, how these transporters respond to water stress during citrus granulation, and whether their expression changes correlate with inter-tissue water redistribution, remain largely unknown.

Previous studies on citrus granulation mainly focused on cell wall and carbohydrate metabolism, with limited attention to inter-tissue water relations and their linkage with tonoplast transport genes. The novelty of this work lies in its attempt to integrate tissue water relations and tonoplast transporter gene expression to dissect the early triggering events of granulation, rather than examining these components separately. This study hypothesizes that postharvest granulation in tight-skinned sweet orange is initiated by a preferential decline in peel water potential that establishes a peel–pulp water potential gradient, and that this is accompanied by osmolyte depletion and by reprogramming of tonoplast aquaporin and sugar/acid transporter expression. In this study, ‘Newhall’ navel orange was used to investigate the incidence of granulation (granulation rate and index) and the associated water physiological changes (water loss rate, water content and water potential of peel and pulp, and inter-tissue water potential difference) during storage. Additionally, the differences in water physiology and the expression of tonoplast aquaporin and sugar-acid transporter genes between peel and pulp tissues at different granulation grades were examined. The aim was to elucidate the relationship between granulation and water physiology in tight-skinned citrus, and to provide a theoretical basis for mitigating this quality disorder through the regulation of water dynamics.

## 2. Materials and Methods

### 2.1. Fruit Materials

In November 2021, approximately 600 kg of mature ‘Newhall’ navel orange (*Citrus sinensis* (L.) Osbeck) fruits were harvested from a local commercial orchard in Ganzhou City, Jiangxi Province, China, and were transported to the laboratory within six hours. After dissipating field heat in a ventilated environment for two days, the fruits with uniform size and free from any visible injury were manually cleaned, air-dried, and individually sealed in polyethylene bags (18 cm × 14 cm × 5 μm), then stored at ambient temperature (8–12 °C, 85–90% RH) for 200 days.

Samples of peel and pulp were randomly collected from 20 fruits at 0, 20, 40, 60, 80, 100, 120, 140, 160, 180 and 200 d during storage, and immediately cooled with liquid nitrogen, then stored at −80 °C for analyzing osmolytes and gene expression.

### 2.2. The Granulation Level, Granulation Rate and Granulation Index

Forty fruits were randomly selected and equally divided into three replicates for granulation level, granulation rate and granulation index. Juice-sac granulation in navel orange expands from the peduncle (calyx) end and the stylar end toward the equatorial region. Granulation severity was classified into four levels based on the proportion of dehydrated juice sacs observed in the longitudinal section of each fruit.

Level 0, no granulation: Granulation area 0;

Level 1, early granulation: granulation area < 1/10;

Level 2, moderate granulation: granulation area 1/10–1/5;

Level 3, severe granulation: granulation area > 1/5.

The rate of granulation was determined using the following formula:

(1) $$\mathrm{Granulation}\;\mathrm{rate}\;(\%)=\frac{\mathrm{Number}\;\mathrm{of}\;\mathrm{granulated}\;\mathrm{fruits}}{\mathrm{Total}\;\mathrm{number}\;\mathrm{of}\;\mathrm{fruits}}\times 100\%$$

The granulation index was determined using the following formula:

(2) $$\mathrm{Granulation index}=\frac{\Sigma (\mathrm{Granulation}\;\mathrm{level}\times \mathrm{fruit}\;\mathrm{number}\;\mathrm{of}\;\mathrm{this}\;\mathrm{level})}{3\times \mathrm{total}\;\mathrm{fruit}\;\mathrm{number}}\times 100\%$$

### 2.3. Weight Loss

Sixty fruits were randomly selected and equally divided into three replicates for weight loss estimation. Fruit weight was individually recorded at each sampling time point. The accumulated weight loss was calculated as the change from initial weight (at 0 d) to the final weight (at each time point). The average weight loss was calculated as the weight loss within each 20-day interval.

The calculation formula is as follows:

(3) $$\mathrm{Accumulated}\;\mathrm{weight}\;\mathrm{loss}\;(\%)=\frac{W0-Wn}{W0}\times 100\%$$

(4) $$\mathrm{Average}\;\mathrm{weight}\;\mathrm{loss}\;(\%)=\frac{Wn-Wn+20}{Wn}\times 100\%$$

Note: The “0” indicates 0 days of storage. The “n” indicates the storage time point. The “W” indicates fruit weight.

### 2.4. Water Content

To determine the water content in peel and pulp, 10-millimeter-wide strips of peel from the equatorial region were excised and two segments were collected from each fruit. Six to seven fruits for each biological replicate and a total of 20 fruits were used. The peel and pulp samples were weighed before and after drying at 80 °C for 96 h. The calculation formula is as follows:

(5) $$\mathrm{Water}\;\mathrm{content}\;(\%)=\frac{\mathrm{fresh}\;\mathrm{weight}-\mathrm{dry}\;\mathrm{weight}}{\mathrm{fresh}\;\mathrm{weight}}\times 100\%$$

### 2.5. Water Potential

The water potential was measured using a PSYPRO water potential system equipped with a C-52 sample chamber and L51 probe (Wescor, South Logan, UT, USA). Six to seven fruits for each biological replicate and a total of 20 fruits were used. As described previously [25], 10-millimeter disks of the fruit peel were excised from the central outer region of the fruit (region with the highest circumference) using a cork borer. These disks were then immediately placed in a sample chamber to measure the water potential of the peel. The juice sacs of orange fruit are fragile, and the pulp water potential cannot be estimated directly. Hence, the juice sacs were squeezed to obtain fruit juice. A piece of filter paper fully saturated with the juice was placed in the sample chamber to measure the water potential of the pulp. The PSYPRO water potential system was previously calibrated with NaCl solutions of known concentrations. To ensure initial water vapor equilibrium, water potential measurements were taken at least 30 min after setting the sample into the chamber.

### 2.6. Osmolytes Measurement

#### 2.6.1. Soluble Sugars

Soluble sugar concentration was determined by the anthrone colorimetric method. Following our published protocol [26], one gram of tissue was homogenized using a Retsch-MM 400 mixer mill apparatus (Retsch Inc.; Newtown Pa., Germany), then mixed with 100 mL distilled water and bathed in boiling water for 30 min. After centrifugation (Hettich-UNIVERSAL 320R, Tuttlingen, Germany) at 8000× *g* for 10 min, 2 mL of 50-fold diluted supernatant was reacted with 0.5 mL of 2% anthrone-ethyl acetate reagent and 5 mL of concentrated sulfuric acid in a boiling water bath for 60 s. The reaction solution was air-cooled to room temperature and the absorbance was then measured at 630 nm by an ultraviolet–visible (UV–vis) spectrophotometer (UV-2600, Shimadzu, Kyoto, Japan). The result was ultimately expressed as mg/g of fresh weight. For each granulation grade, nine fruits were randomly selected and evenly allocated into three replicates to measure soluble sugar content.

#### 2.6.2. Soluble Protein

Soluble protein concentration was assayed by the Coomassie brilliant blue G-250 binding method. Two grams of flesh tissue were homogenized using a Retsch-MM 400 mixer mill apparatus (Retsch Inc.; Newtown Pa., Germany), then mixed with 5.0 mL phosphate buffer. After centrifugation (Hettich-UNIVERSAL 320R, Tuttlingen, Germany) at 12,000× *g* for 20 min at 4 °C, 1.0 mL of supernatant was reacted with 5.0 mL Coomassie brilliant blue G-250 solution for 2 min. The absorbance value of the reaction solution was detected at 595 nm by an ultraviolet–visible (UV-Vis) spectrophotometer (UV-2600, Shimadzu, Kyoto, Japan). Soluble protein concentration was calculated from a standard curve, and the results were expressed as mg·g^−1^ fresh weight. For each granulation grade, nine fruits were randomly selected and evenly allocated into three replicates to measure soluble protein.

#### 2.6.3. Components of Sugars and Organic Acids

Referring to our previous methods [27] for sugar and organic acid detection, 4 g of frozen powder sample was water-bathed with 25 mL of 80% ethanol at 35 °C for 20 min, then centrifuged at 10,000× *g* for 15 min. One milliliter of supernatant was evaporated at 30 °C until gelatinous using a rotary evaporator (5305, Eppendorf AG 122,339 Hamburg, Germany), then re-dissolved in 1 mL of ultrapure water. After being filtered through a 0.45 μm or 0.22 μm film, the sugars or acids in the solution were quantified using HPLC (LC-20A, Shimadzu, Japan) equipped with an RID detector or diode array detector. The sample volume was 20 μL. The chromatographic conditions for sugar detection were: Waters NH_2_ column (4.6 mm × 250 mm, 5.0 μm), column temperature kept at 35 °C; mobile phase, acetonitrile: water (85: 15) at a rate of 0.8 mL·min^−1^. The chromatographic conditions for organic acid detection were: Waters C18 column (4.6 mm × 250 mm, 5.0 μm), column temperature kept at 25 °C; mobile phase, 0.01 mol·L^−1^ H_2_SO_4_ (using KOH to adjust the pH to 2.6) at a rate of 0.5 mL·min^−1^. The concentration of sugar and acid in the sample was determined based on the standard curve derived from the sugar and acid standards, and was ultimately expressed as mg·g^−1^ of fresh weight. For each granulation grade, nine fruits were randomly selected and evenly allocated into three replicates to measure sugars and organic acids.

#### 2.6.4. Mineral Elements

About 0.5 g of dry sample was weighed in a 25 mL ceramic crucible, and baked in a muffle furnace at 550 °C for 5 h. The ash was taken out slightly and cooled to room temperature, then mixed with 10 mL of acid solution (60% water, 30% hydrochloric acid, 10% nitric acid); 30 min later, the filtrate was directly detected by ICP-OES5110 (Agilent, America) ICP-AES. The calculation formula is: fresh weight concentration of mineral elements = dry weight concentration of elements/(100–tissue water content %). The result was ultimately expressed as mg·g^−1^ of fresh weight. For each granulation grade, six to nine fruits were randomly selected and evenly allocated into three replicates to measure mineral elements.

### 2.7. RNA Extraction, cDNA Synthesis and qRT-PCR

About 2 g of peel and pulp tissues were used to extract total RNA with TRIzol reagent according to the procedure of the previous report [27] and three biological replicates were performed. One microgram (μg) of high-quality total RNA was used for first-strand cDNA synthesis using a PrimeScript RT Reagent kit with a gDNA Eraser (TaKaRa, Dalian, China). qRT-PCR was performed in a 10 µL reaction volume using the SYBR Premix Ex Taq II Kit (TaKaRa, Dalian, China) in a Bio-Rad CFX96 (Bio-rad, Hercules, CA, USA), with cycling conditions of 95 °C for 30 s followed by 40 cycles of 95 °C for 5 s and 60 °C for 30 s; the relative expression was calculated by the 2^−ΔΔCt^ method with three biological replicates and three technical replicates each. Primer sequences were synthesized in accordance with our published paper [28] and Table S1 in the Supplementary Material.

### 2.8. Statistical Analysis

ANOVA (Duncan’s multiple-range test at *p* < 0.05), T-test, Pearson’s correlation analysis, and principal component analysis (PCA) were performed with SPSS Statistics 26.0 software (IBM SPSS Inc., Chicago, IL, USA) at the level of *p* < 0.05. Normality was evaluated by descriptive statistics (mean, standard deviation, maximum and minimum values). Levene’s test was used to assess homogeneity of variances. One-way analysis of variance (ANOVA) was conducted for data that approximately met normality and homogeneity assumptions. Duncan’s multiple range test was used for post-hoc multiple comparisons, and differences were regarded as significant at *p* < 0.05. Plots were graphed by GraphPad Prism 8.0 software (GraphPad, San Diego CA, USA). Gene expression cluster plots were conducted by TBtools 2.0 [29]. Gene expression and physiological indicators associated with granulation, Pearson’s correlation analysis was performed using the online ChiPlot software (https://www.chiplot.online/, accessed on 21 September 2026). All data are expressed as the mean ± standard deviation (SD).

## 3. Results

### 3.1. Temporal Dynamics of Granulation in Postharvest ‘Newhall’ Navel Orange Fruits

**Figure 1:**
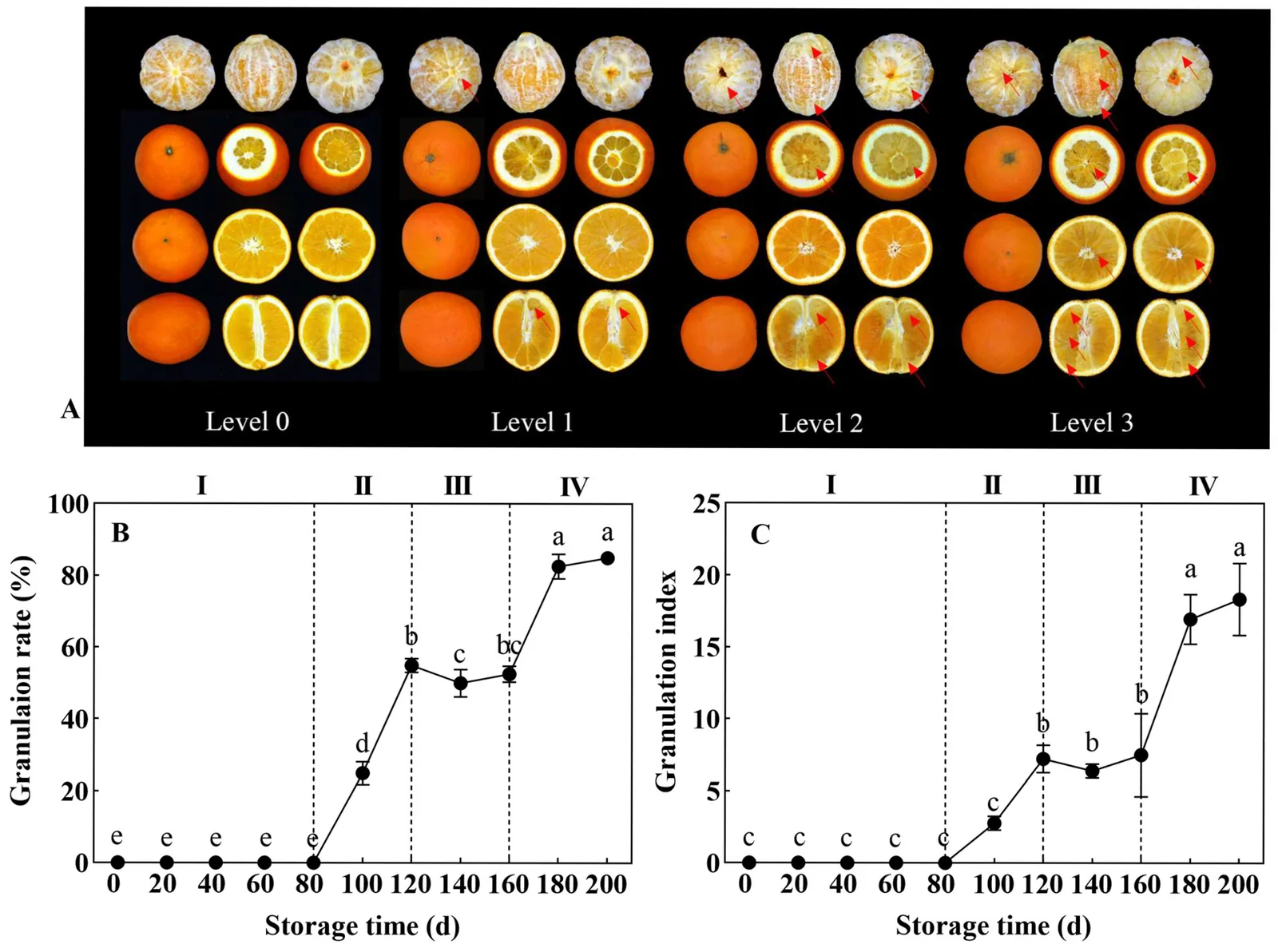
Phenotypic photographs of ‘Newhall’ navel orange fruits with different degrees of granulation (**A**), and the granulation rate (**B**) and granulation index (**C**) during 200 days of storage under ambient conditions. Note: The red arrow marks the location of the granulation disease onset in (**A**). The values are presented as means ± SD. Different lowercase letters indicate significant differences (Duncan, *p* < 0.05) among different storage times within the same tissue. The following figures were the same.

Granulation development was monitored in ‘Newhall’ navel oranges over a 200-day ambient-temperature storage period. Granulation degrees were classified into four levels based on severity (Figure 1A). Notably, the external appearance of the fruit peel showed no visible changes during granulation; however, the pulp exhibited pronounced degeneration, characterized by color fading and dehydrated, shriveled, and hardened juice sacs.

No granulation symptoms were observed within the first 80 days postharvest. The granulation rate and granulation index increased sharply between 80 and 120 days, reaching 55.95% and 7.22, respectively, by day 120, after which they showed no significant increase until day 160. A second increase occurred between 160 and 180 days, with the values ultimately reaching 84.98% and 18.33 by day 200 (Figure 1B,C).

Based on the postharvest granulation progression of ‘Newhall’ navel orange, the entire storage period can be divided into four stages: I, 0–80 d, healthy to sub-healthy stage; II, 80–120 d, granulation initiation and development stage; III, 120–160 d, granulation aggravation stage; and IV, 160–200 d, severe granulation stage.

### 3.2. Temporal Dynamics of Water Physiological Indexes During the Granulation Progress in Postharvest ‘Newhall’ Navel Orange Fruits

**Figure 2:**
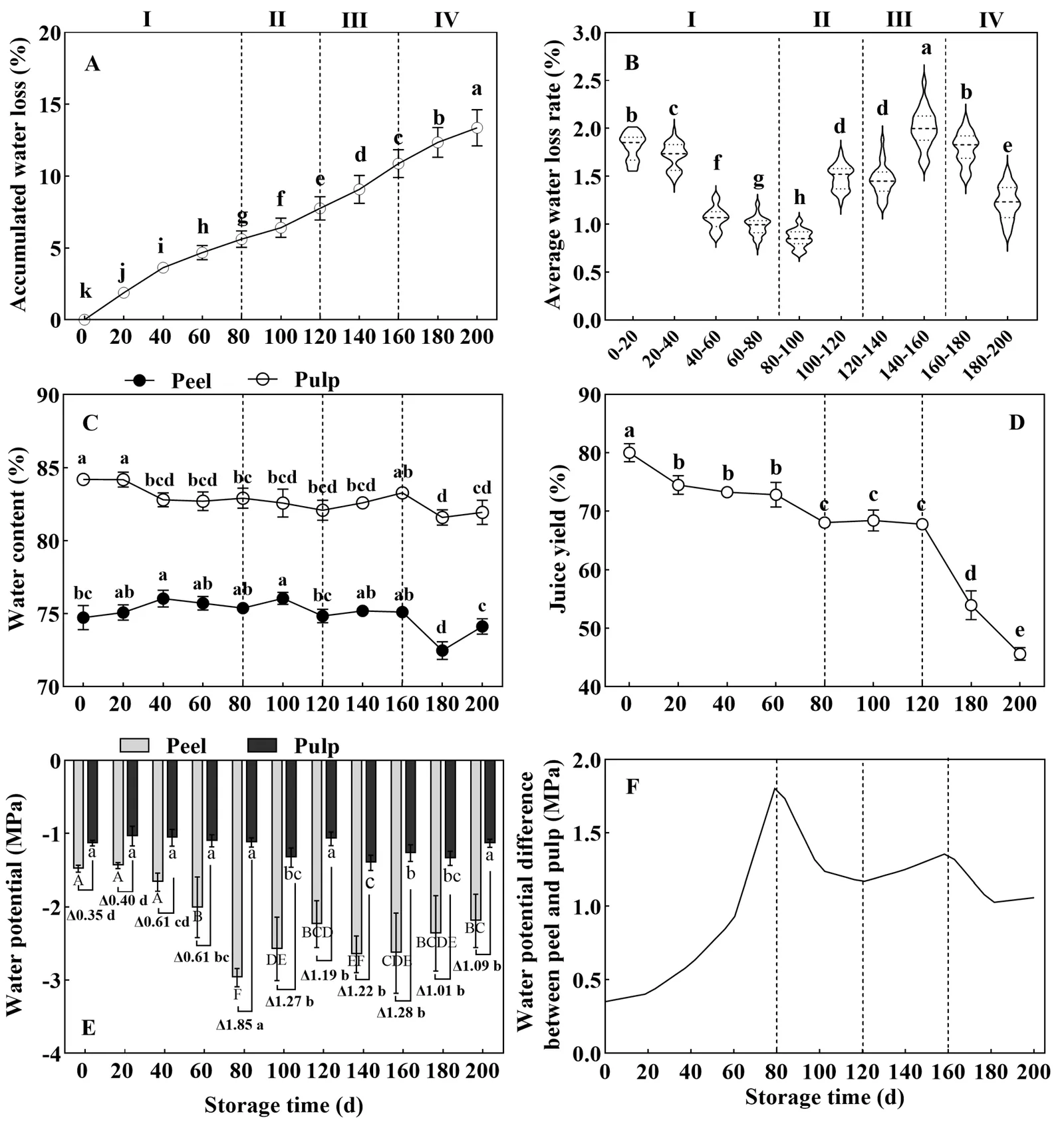
Changes in fruit weight loss (**A**,**B**), water content (**C**), juice yield (**D**), water potential (**E**), and peel–pulp water potential difference (**F**) of navel orange during ambient storage, in relation to granulation stages. Note: Data are presented as mean ± SD, with n ≥ 3. Different uppercase letters and lowercase letters indicate significant differences during different storage times within the same tissue. Error bars represent the standard deviation (SD); small error bars indicate low biological variation among replicates.

Postharvest water loss in Newhall navel oranges continues to increase, but not at a constant rate during the granulation progress (Figure 2A,B). In the healthy to sub-healthy stage I, the average weight loss rate per 20 days was higher initially (0–40 d) but constantly decreased to the lowest till 80 d; for the granulation initiation and aggravation stages of II and III, it was recovered and reached its highest at the period of 140–160 d; in the severe granulation stage of IV, it dropped slightly and kept at a higher speed till 200 d. Additionally, the accumulated water loss of granulated fruit was significantly higher than that of normal fruits (Supplemental Figure S1A,B). The results suggest that water loss accelerates after the onset of granulation.

The water content of the pulp (81.59–84.19%) was consistently higher than that of the peel (72.47–76.04%). In stage I, the water content of the peel increased slightly from 74.73% to 76.04%, while that of the pulp decreased from 84.19% to 82.90%. Different from the trends of water loss rate, in stages II and III, the water content of both the peel and the pulp remained relatively stable. Entering stage IV, especially during the period of 160–180 d, the water content of both the peel and the pulp decreased sharply from 75.11% to 72.47% in the peel, and from 83.28% to 81.59% in the pulp (Figure 2C). Similarly, the change in juice yield of the pulp followed a trend consistent with that of water content (Figure 2D).

The water potential difference serves as the driving force for water movement between tissues. The water potential of the pulp remained consistently higher than that of the peel throughout the storage period (Figure 2E). During granulation progression, pulp water potential remained stable in stage I but decreased significantly in stages II, III, and IV after the onset of granulation (at 100, 140, 160, and 180 d, respectively). In contrast, peel water potential remained stable during the early part of stage I (within the first 40 d) but decreased rapidly thereafter, reaching its minimum value of −2.96 MPa at the onset of granulation (80 d). It subsequently increased to −2.24 MPa in stage II, then decreased again to −2.63 MPa in stage III, and remained at a relatively low level (−2.19 MPa) by stage IV (200 d).

The water potential difference between the peel and pulp (Figure 2F) was greatest at the onset of granulation, indicating rapid water transport from the pulp to the peel. After the initiation of granulation (stage II), the water potential difference decreased markedly; however, it remained relatively large during the granulation development stage (stage III). In contrast, the water potential difference gradually diminished during the severe granulation stage (stage IV).

These water physiological changes during granulation development suggest that a sharp decline in peel water potential and a rapid increase in weight loss rate serve as key indicators of granulation initiation.

### 3.3. Water-Related Indicators Across Different Granulation Levels in Peel and Pulp Tissues

#### 3.3.1. Water Content and Water Potential of Peel and Pulp at Different Granulation Grades

To investigate the relationship between granulation severity and water physiological changes, peel and pulp tissues of different granulation grades were collected from fruits stored for 180 days. The water content, water potential, and the levels of osmotic regulatory substances, including sugars, organic acids, soluble proteins, proline, and inorganic ions, were determined.

**Figure 3:**
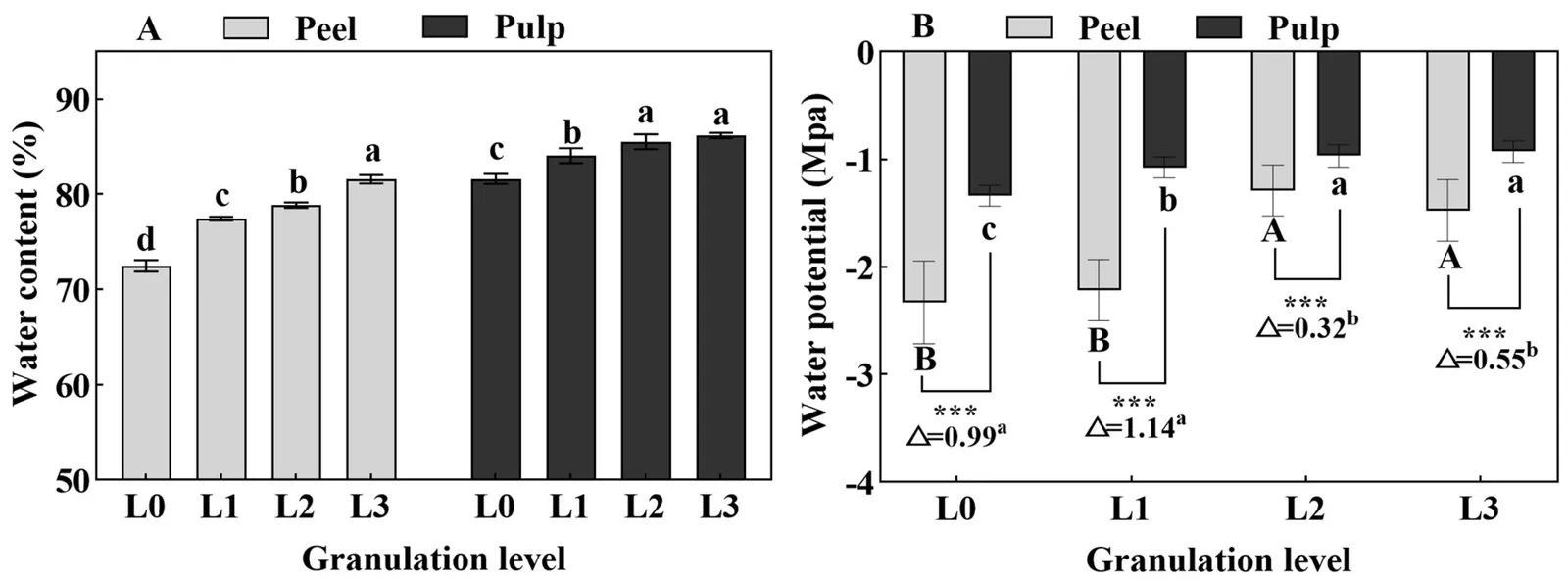
Water content (**A**) and water potential (**B**) at different granulation levels. Note: Data are presented as mean ± SD, with n ≥ 3. Different uppercase letters and lowercase letters indicate significant differences during different granulation grades within the same tissue. “***” represent the significance at the levels of *p* < 0.001.

With increasing severity of granulation, the water content and water potential of both peel and pulp tissues showed an overall upward trend (Figure 3A,B). Significant differences were observed in peel water potential between grades L1 and L2, and in pulp water potential among grades L0, L2, and L3 (Figure 3B). The water potential gradient difference between the peel and pulp also exhibited large differences at stages L0 and L1 (0.99 MPa and 1.14 MPa), but smaller differences at stages L2 and L3 (0.32 MPa and 0.55 MPa). These results indicate that elevated water content and water potential difference are important conditions for the occurrence and development of granulation (stage L2), whereas no significant changes occur after severe granulation has developed.

#### 3.3.2. Organic and Inorganic Osmotic Regulatory Substances in Peel and Pulp at Different Granulation Grades

**Figure 4:**
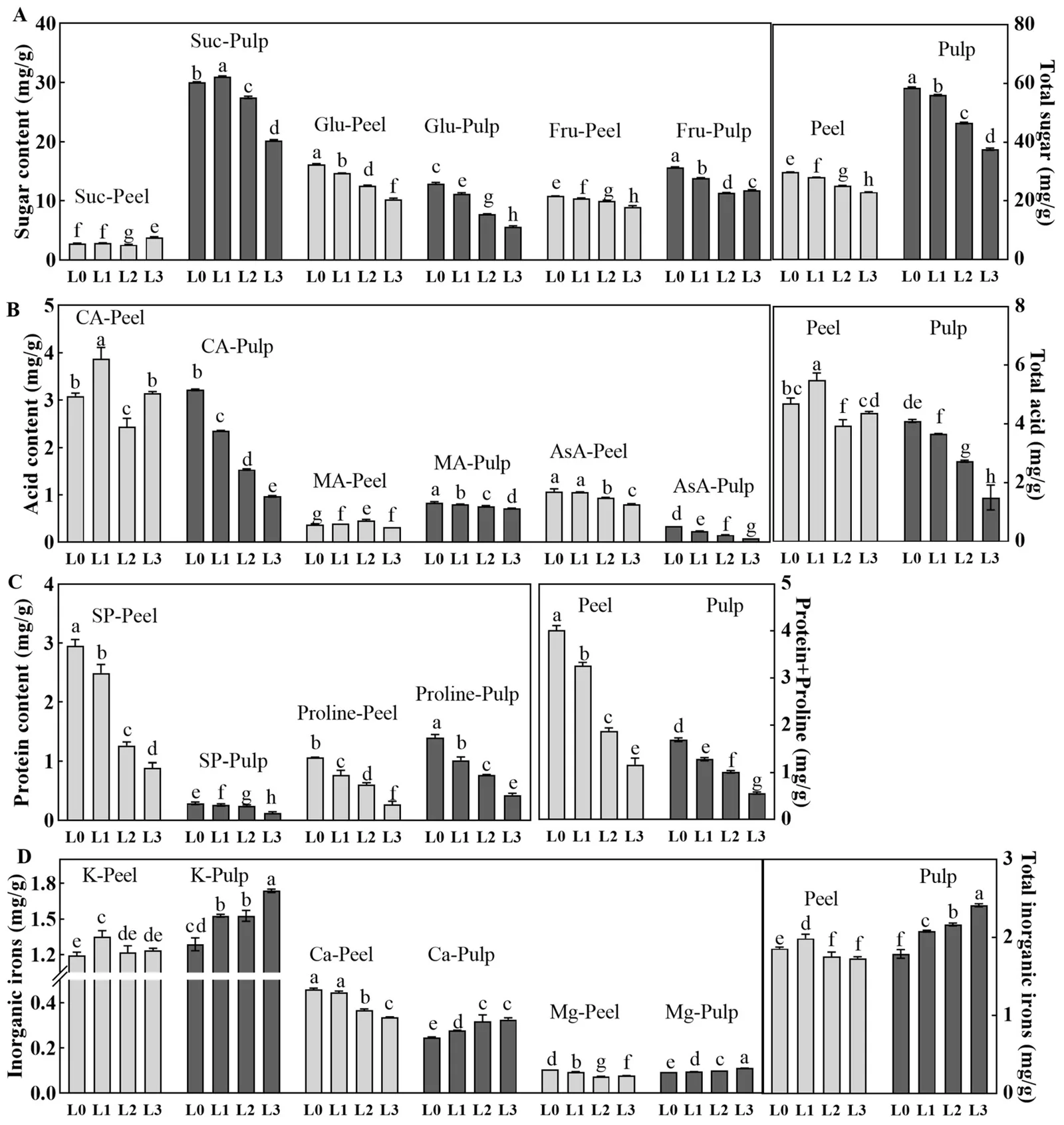
The changes in osmotic regulatory substances, including sugars (**A**), organic acids (**B**), soluble proteins and proline (**C**), and inorganic ions (**D**) in the peel and pulp at different granulation levels. Note: Suc, sucrose; Fru, fructose; Glu, glucose; CA, citric acid; MA, malic acid; AsA, ascorbic acid; SP, soluble protein. One-way analysis of variance (ANOVA) was performed, and mean values were separated using Duncan’s multiple range test. Values with different lowercase letters are significantly different at *p* < 0.05 among different granulation grades, peel and pulp tissues.

Regardless of granulation severity, the contents of fructose, sucrose, malic acid and proline were consistently higher in the pulp than in the peel, whereas the peel exhibited higher levels of glucose, citric acid, and ascorbic acid. Among organic osmotic regulators, total sugar content in the pulp was 1.64- to 2.00-fold higher than that in the peel, while total organic acids and total proteins in the peel were 1.14- to 2.92-fold and 5.14- to 10.23-fold higher than those in the pulp, respectively (Figure 4A–C). The main mineral elements were potassium (K), calcium (Ca) and magnesium (Mg). Pulp K and Mg contents were 1.07- to 1.40-fold and 6.67- to 7.55-fold higher than those in the peel, respectively, whereas peel Ca content was 1.03- to 1.87-fold higher than that in the pulp (Figure 4D). Overall, the contents of osmotic regulators were higher in the pulp than in the peel. During granulation progression, most osmotic regulators in both tissues exhibited a declining trend, with the exception of mineral elements in the pulp, which increased with increasing granulation severity (Figure 4D). Notably, however, citric acid and K in the peel showed a distinct pattern: both peaked at grade L1 and then decreased markedly at grades L2 and L3.

### 3.4. Expression Dynamics of CsTIPs, Tonoplast-Localized Sugar and Acid Transporter Genes in Orange Fruit During Juice Vesicle Granulation

#### 3.4.1. Expression Profiles of CsTIPs in the Peel and Pulp at Different Granulation Grades

**Figure 5:**
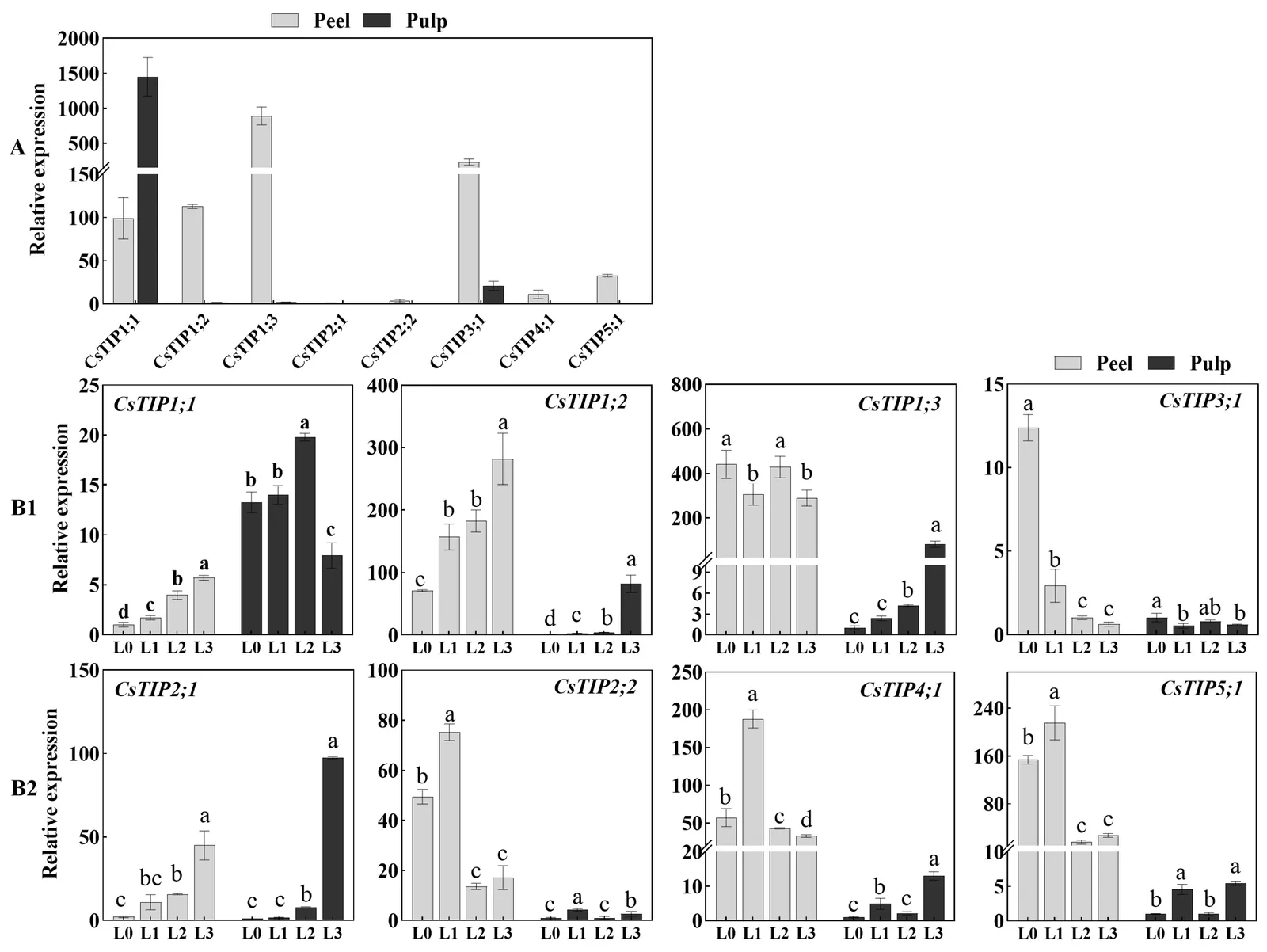
Expression profiles of *CsTIPs* in citrus fruit during granulation. (**A**) The relative expression levels of *CsTIP* members in healthy fruit were calculated based on the expression level of *CsTIP2;1* in the peel at the L0 stage of granulation, enabling inter-member comparison. (**B**) The relative expression levels of highly (**B1**) and lowly (**B2**) expressed *CsTIP* members in the peel and pulp across different granulation stages. The expression level of *CsTIP1;1* was normalized to its value in the peel at L0, while the other members were normalized to their respective L0 values in the pulp. Because of the different baselines, expression values should not be compared directly between panels (**A**,**B**). Note: Data are presented as mean ± SD, with n ≥ 3. Different lowercase letters indicate significant differences during different granulation grades within the same tissue.

In healthy fruit, members of the CsTIP family displayed distinct tissue-specific expression patterns (Figure 5A). In the peel, *CsTIP1;1*, *CsTIP1;2*, *CsTIP1;3*, and *CsTIP3;1* were all highly expressed, with *CsTIP1;3* showing the highest abundance (relative expression level: 891.22). In contrast, only *CsTIP1;1* exhibited substantial expression in the pulp, with a relative expression level of 1451.53, making it the most abundant member in this tissue. The remaining genes, *CsTIP2;1*, *CsTIP2;2*, *CsTIP4;1*, and *CsTIP5;1*, were expressed at relatively low levels in both peel and pulp.

As granulation severity increased, the highly expressed *CsTIP1;1* and *CsTIP1;2* in the peel were continuously upregulated, whereas *CsTIP1;3* and *CsTIP3;1* were downregulated. At grade L3, their expression levels reached 5.71-, 3.98-, 0.56-, and 0.06-fold of those at grade L0, respectively. In the pulp, *CsTIP1;1*, the most abundant member, was upregulated by 1.27-fold at grade L2 but declined to 0.53-fold at grade L3.

Notably, several low-abundance *TIP* genes were significantly upregulated during granulation progression. In the peel, *CsTIP2;1* was upregulated 20.64-fold at grade L3, while *CsTIP2;2*, *CsTIP4;1*, and *CsTIP5;1* were upregulated 1.52-, 3.28-, and 1.41-fold at grade L1, respectively, followed by downregulation at grades L2 and L3. In the pulp, *CsTIP1;2*, *CsTIP1;3*, *CsTIP2;1*, *CsTIP4;1*, and *CsTIP5;1* were upregulated to 81.87-, 57.16-, 97.46-, 12.98-, and 5.47-fold of the L0 level at grade L3, suggesting that the massive induction of these low-abundance aquaporins may be closely associated with the progression of granulation in the pulp. Collectively, these results indicate that *CsTIP1;1*, *CsTIP1;2*, and *CsTIP1;3* are the principal TIP members mediating water transport during granulation, and that the overall abundance of aquaporin transcripts in the peel was substantially higher than that in the pulp.

#### 3.4.2. Expression Dynamics of Tonoplast-Localized Sugar and Acid Transporter Genes in the Peel and Pulp at Different Granulation Grades

**Figure 6:**
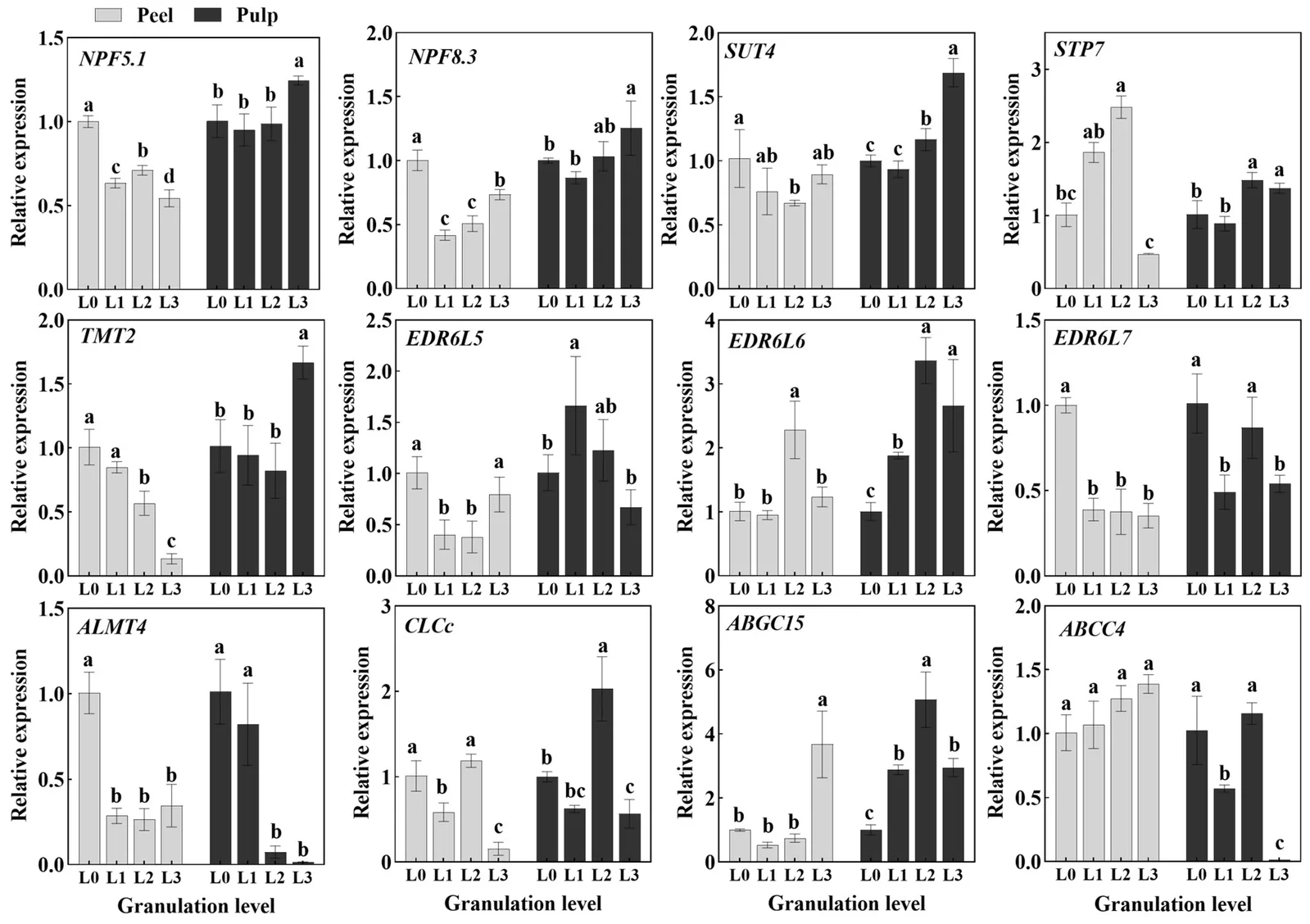
Expression dynamics of tonoplast-localized sugar and acid transporters in citrus fruit during juice vesicle granulation. Note: Data are presented as mean ± SD, with n ≥ 3. Different lowercase letters indicate significant differences during different granulation grades within the same tissue.

Following granulation, most tonoplast sugar transporters—including *NPF5.1*, *NPF8.3*, *SUT4*, *TMT2*, *EDR6L5*, and *EDR6L7*—were significantly downregulated in the peel, except for *STP7* and *EDR6L6*, which were upregulated by more than 2-fold at grade L2. The acid transporters *ALMT4* and *CLCc* were downregulated, whereas *ABGC15* was upregulated 3.67-fold at grade L3, and *ABCC4* showed no significant change (Figure 6).

In the granulated pulp, the sugar transporters *NPF5.1*, *NPF8.3*, *SUT4*, *STP7*, *TMT2*, *EDR6L5*, and *EDR6L6* (1.88- to 3.36-fold) and the acid transporter *ABGC15* (2.81- to 5.56-fold) were significantly upregulated at grade L2 and/or L3. In contrast, the sugar transporter *EDR6L7*, the acid transporters *CLCc* and *ABCC4* (at L1 and/or L3), and *ALMT4* (at L2 and/or L3) were downregulated.

### 3.5. Expression Pattern of Tonoplast Transporter Genes

**Figure 7:**
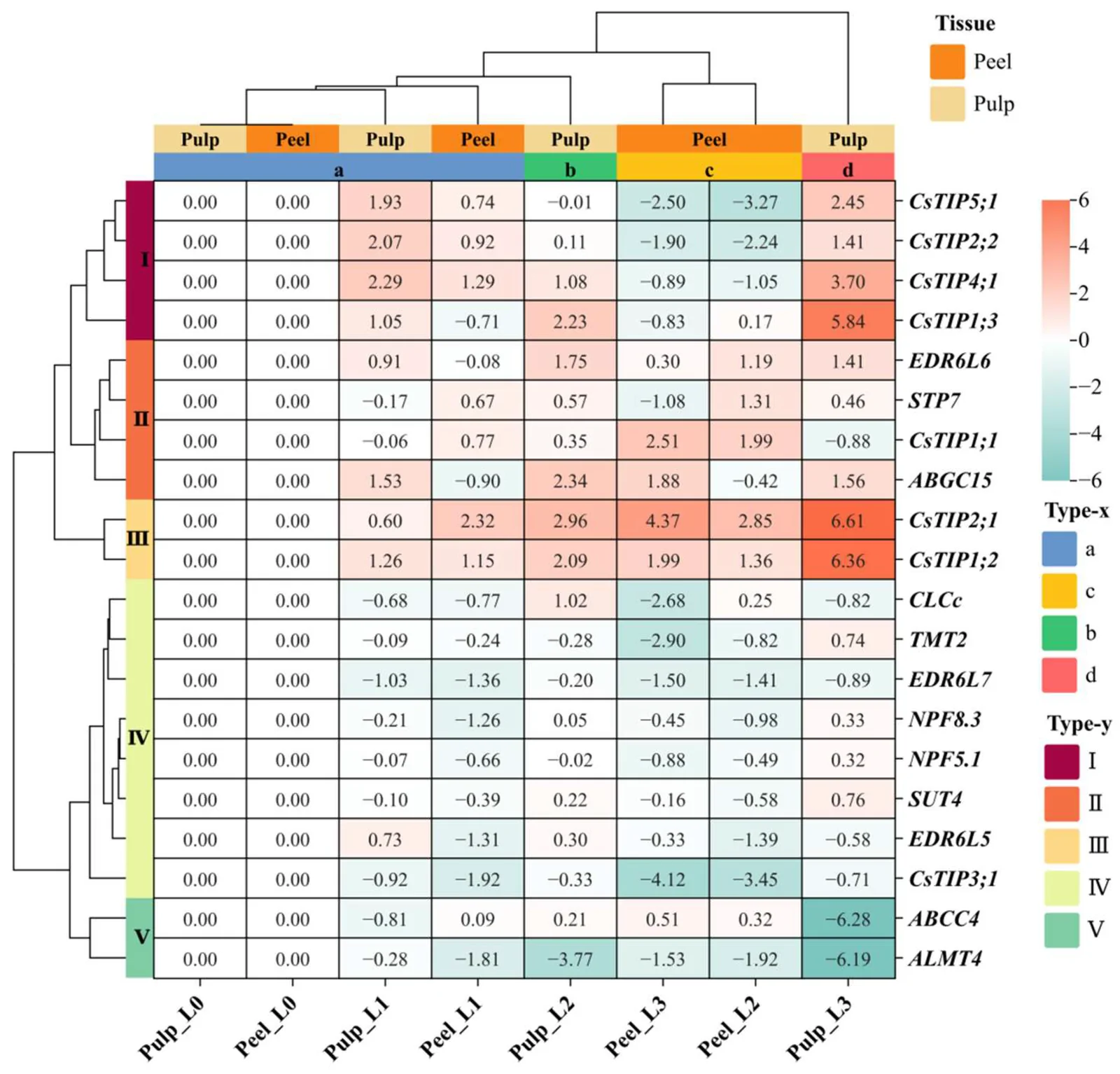
Hierarchical clustering of vacuolar membrane transporter genes (sugar/acid transporters and CsTIPs) based on their expression patterns in peel and pulp tissues across different granulation grades. Note: Pearson’s correlation between relative expression, with differential expression in the clustering analysis defined as |Log_2_FC| > 1.2 at *p* < 0.05.

To characterize the overall expression patterns of 20 vacuolar membrane transporter genes (including sugar and acid transporters and CsTIPs) in response to granulation, hierarchical clustering analysis was performed based on their relative expression levels in both peel and pulp tissues across four granulation grades (L0, L1, L2, and L3) (|Log2FC| > 1.2, *p* < 0.05) (Figure 7). The eight tissue-grade samples were grouped into four distinct clusters. The L0 and L1 samples from both peel and pulp formed the first cluster (type a). Peel-L2 and peel-L3 constituted the third cluster (type c), whereas pulp-L2 and pulp-L3 fell into the second and fourth clusters (types b and d), respectively.

Vertically, the 20 differentially expressed genes were grouped into five clusters. Group I comprised *CsTIP1;3*, *CsTIP2;2*, *CsTIP4;1*, and *CsTIP5;1*, which showed consistent downregulation in peel but upregulation in pulp. Group II consisted of EDR6L6, STP7, *CsTIP1;1* and *ABGC15*, all of which exhibited sustained upregulation specifically in both peel and pulp at L1 and L2. Group III included *CsTIP1;2* and *CsTIP2;1*, showing consistent upregulation in both tissues. Group IV encompassed *CLCc*, *TMT2*, *EDR6L7*, *NPF5.1*, *NPF8.3*, *SUT4*, *EDR6L5* and *CsTIP3;1*, all of which were downregulated in the peel. Group V, comprising *ABCC4* and *ALMT4*, showed downregulation in both peel and pulp, particularly at L3. These vacuolar membrane transporter genes, which were specifically upregulated in distinct tissues and at different granulation stages, may be involved in water movement within or between tissues during the progression of granulation.

### 3.6. Correlation Analysis

**Figure 8:**
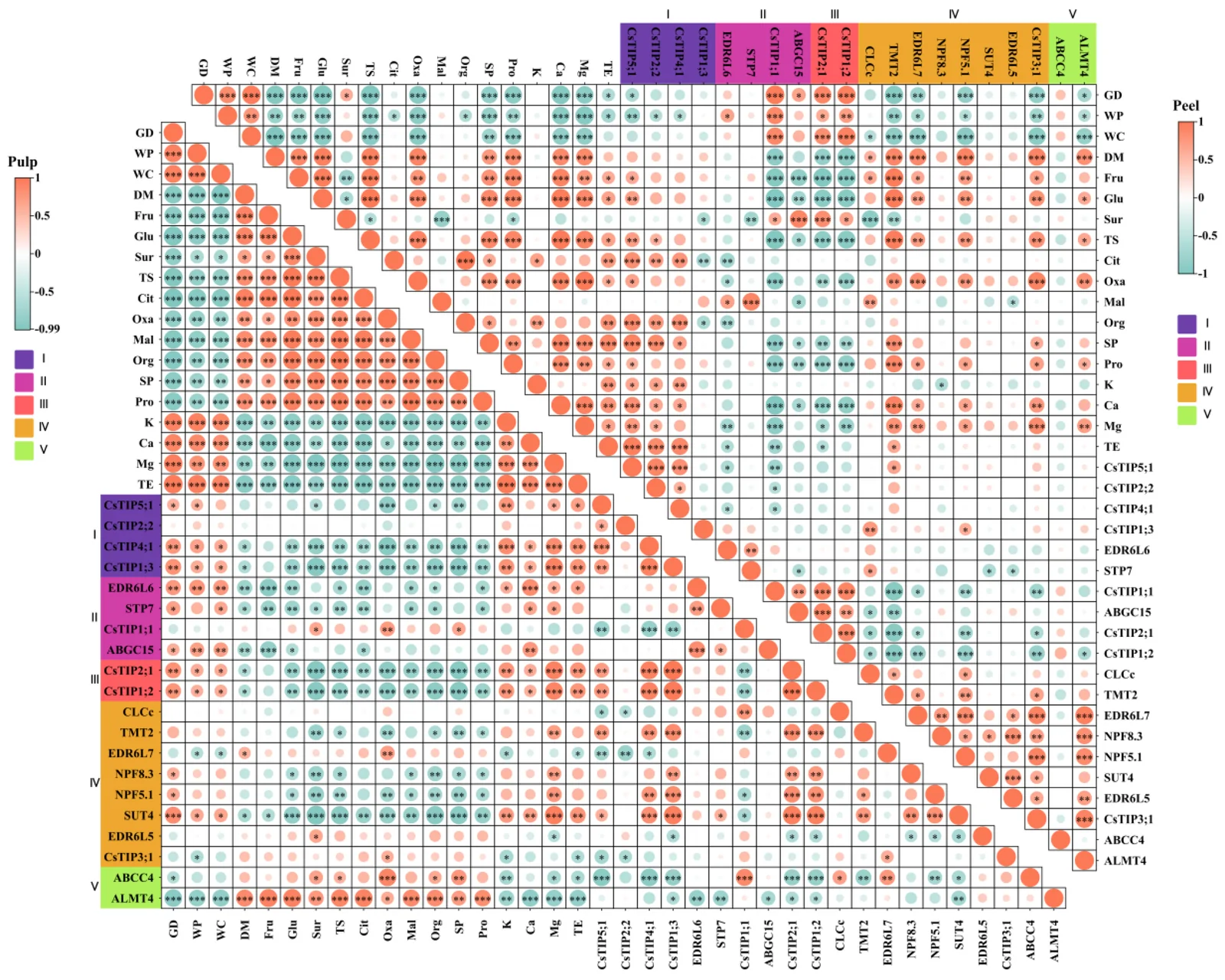
Pearson’s correlation of AQPs, sugar and acid vacuole membrane transporter gene expression with osmolytes and water-related indexes in the peel and pulp of fruit. Note: GD, granulation degree; WP, water potential; WC, water content; DM, dry matter; Fru, fructose; Glu, glucose; Suc, sucrose; TS, Total sugar; Cit, citric acid; Oxa, oxalic acid; Mal, malic acid; Org, organic acid; SP, soluble protein; Pro, proline; TE, total element. “*”, “**” and “***” represent the significance at the levels of *p* < 0.05, 0.01 and 0.001, respectively. The orange-red or green circle represents the positive or negative co-relationship with ∣R∣ > 0.8.

The relationships between granulation degree and osmolyte-related physiological indicators as well as vacuole membrane transporter gene expression, in the samples of L0 to L3, were further analyzed using Pearson’s correlation method (Figure 8). Both in the peel and pulp, it exhibited positive correlations between granulation degree and water potential, water content, transporter gene expression of *ABGC15* (in group II) and *CsTIP1;2/2;1* (in group III), while showing negative correlations with dry matter, fructose, glucose, total sugar, oxalic acid, soluble protein, proline and *ALMT4* genes (in group V). The granulation severity in the peel was negatively correlated with the contents of Ca, Mg, and total mineral elements, whereas that in the pulp was positively correlated with the contents of K, Ca, Mg, and total mineral elements.

## 4. Discussion

### 4.1. Preferential Decline in Peel Water Potential Drives the Initiation of Granulation

The present study demonstrated that the initiation of postharvest granulation in Newhall navel orange (*Citrus sinensis* Osbeck) began with a sharp decline in peel water potential, thereby establishing a reversed water potential gradient between the peel and pulp that drove water migration from the pulp to the peel. At 80 days of storage, the peel water potential had decreased to −2.96 MPa, and the peel–pulp water potential difference peaked at 1.85 MPa, coinciding with the rapid increase in the granulation index (Figure 1B,C and Figure 2E,F). In addition, cumulative weight loss reached approximately 8% before 80 days of storage (Figure 2A,B), whereas the granulation index increased rapidly after 80 days, implying that whole-fruit rapid water loss is closely related to the process of granulation. These data are, however, largely correlative: the temporal coincidence of the water potential decline with granulation does not by itself establish causality. We therefore interpret the reversed gradient as a plausible physical driving force for water migration rather than a demonstrated cause, and note that causal inference would require manipulative experiments. In line with these findings, the use of a ^3^H_2_O tracer confirmed that the rate of water transfer from the pulp to the peel in granulated fruit was significantly higher than that in normal fruit [30]. Similarly, significant changes were observed in inter-tissue water potential gradients during storage in multiple citrus types [14]. Such observations strengthen the association but do not prove a causal role.

However, why does the peel water potential decline preferentially? The “cell wall metabolism disorder hypothesis” proposed provides a plausible explanation: pectin demethylesterification and lignin accumulation in peel cell walls reduce the water-holding capacity of the cell wall, directly leading to a decline in water potential [31]. And, because cell wall material accounts for a large fraction of peel tissue, it would be expected to lower peel water potential preferentially. This provides a direct link to the physiological data: reduced cell wall water-holding capacity is consistent with the sharp decline in peel water potential (to −2.96 MPa) and with the approximately 8% cumulative weight loss before 80 days of storage, and the resulting reversed gradient would favor net water migration from the pulp to the peel. At the molecular level, the coordinated regulation of membrane transporters—including tonoplast intrinsic proteins, tonoplast sugar/acid transporters—modulates vacuolar water permeability and osmotic potential, and thus the water-retention capacity of peel and pulp cells. Cell wall remodeling and vacuolar transport therefore are hypothesized to act in concert to establish the preferential decline in peel water potential.

### 4.2. Osmolyte Depletion Exacerbates Water Imbalance

Water loss from the pulp driven by the water potential gradient further triggered dysfunction of the osmotic adjustment system. Soluble sugars and organic acids are core solutes for maintaining cellular osmotic potential, and their depletion directly reduces the water-holding capacity of juice sacs. In this study, as granulation intensified, most organic osmolytes (sucrose, fructose, citric acid, soluble proteins, and proline) in both the pulp and peel showed fluctuating downward trends (Figure 4A–C); only citric acid and K^+^ in the peel increased slightly at the L1 stage, although the overall trend remained one of depletion. A significant decrease in soluble sugars and organic acids during granulation has likewise been reported in Huyou (*Citrus changshanensis*) [32], and decreases in sugar and acid contents have been shown to be positively correlated with fruit water loss during postharvest storage of citrus [33]. Notably, osmolyte contents were consistently higher in the pulp than in the peel, and this tissue-specific distribution serves as the basis for maintaining water homeostasis in the fruit. This overall decline requires distinguishing between two processes, consumption and reallocation. Citric acid and the nitrogenous osmolytes (soluble proteins and proline) declined in both tissues (Figure 4A–C) and are therefore considered to be consumed, whereas sucrose and fructose appear to be reallocated, their carbon skeletons being diverted from osmotic storage to the synthesis of structural cell wall components. Granulated juice sacs accumulate cellulose, lignin, and hemicellulose while their soluble sugar content declines [31], and in pummelo the sharp decrease in sucrose occurred only after extensive mitochondrial proliferation in the juice sacs, indicating that carbon-source sugars were repartitioned toward cell wall synthesis [34]; it has also been proposed that some sugar components are reallocated owing to cell wall metabolism rather than simply consumed during granulation [31]. This distinction is critical, because reallocation converts osmotically active sugars into osmotically inert cell wall polymers and thus removes them from the osmotic pool as effectively as direct degradation. The strong up-regulation of *CsTIP1;2* and *CsTIP2;1* (81.87- and 97.46-fold at the L3 stage) is consistent with increased tonoplast water permeability as reported in prior functional studies (Figure 5), so that vacuolar water was released into the cytoplasm and, under the prevailing gradient, ultimately lost to the peel.Concurrently, Tonoplast transporter profiling provides correlative molecular evidence consistent with this solute loss: *SUT4* was up-regulated together with *NPF5.1*, *NPF8.3*, *STP7*, *TMT2*, *EDR6L5*, and *EDR6L6* (1.88–3.36-fold at the L2/L3 stages), whereas *ALMT4* and *ABCC4* were down-regulated (Figure 6), so that vacuolar sucrose was drained and organic acid storage impaired, leaving the central vacuole progressively depleted of the solutes that lower its osmotic potential and retain water.

### 4.3. Passive Accumulation of Mineral Elements and Ca^2+^-Mediated Pectin Gelation

Water loss and osmolyte depletion were also accompanied by abnormal redistribution of mineral elements. In this study, as granulation intensified, the K, Ca, and Mg contents in the peel decreased continuously, whereas those in the pulp increased significantly (Figure 4D). However, the accumulation of mineral elements in the pulp did not effectively prevent water loss, indicating that this accumulation was not an active osmotic adjustment but rather a passive concentration process [35]. The following mechanism may account for this phenomenon: peel senescence impairs membrane integrity, causing the leakage of intracellular ions, which then migrate with water to the pulp under the peel–pulp water potential gradient; after water evaporation, these ions are passively concentrated in the pulp. Mineral ions can be passively transported along water flow via the apoplastic pathway without consuming metabolic energy, providing theoretical support for this hypothesis [36].

Tonoplast transporter expression profiles provide a correlative molecular context for these cation dynamics. The tonoplast transporter profiles mirror these cation dynamics: ABCC4, ALMT4, NPF5.1, and NPF8.3 were down-regulated in the peel and up-regulated in the pulp (Figure 6). These transporters move citrate and sugars rather than K^+^, Ca^2+^, or Mg^2+^, but their coordinated reprogramming indirectly governs cation partitioning among the vacuole, the cytosol, and the apoplast by setting the osmotic and electrochemical gradients. Vacuolar cation transport relies on the same proton gradient: CAX exchangers regulate apoplastic Ca^2+^ so as to optimize cell wall expansion [37], CAX1-mediated Ca^2+^ compartmentation directly controls apoplastic Ca^2+^ and cell wall extensibility [38], NHX1/NHX2 antiporters build the vacuolar K^+^ pool and regulate turgor [39], and NPF members themselves act as H^+^/K^+^ antiporters [40], with potassium, chloride, and sugars among their substrates [41]. Down-regulation of *ABCC4/ALMT4* in the peel therefore shrinks the vacuolar solute pool that sustains cation sequestration, favouring cation release once membranes fail, while up-regulation of sugar transporters in the pulp is consistent with the pulp acting as the sink for solutes and ions displaced from the peel. This redistribution also places Ca^2+^ in a particularly critical position. In this study, the abnormal increase in Ca^2+^ in the pulp was closely related to cell wall degradation, as inferred from co-occurring physiological changes. It has been confirmed in prior work on pomelo fruit that released Ca^2+^ re-crosslinks with demethylesterified pectin to form an “egg-box” gel structure, leading to juice sac hardening [42]. An appropriate amount of Ca^2+^ is essential for maintaining cell wall structural integrity, whereas abnormally elevated concentrations instead disrupt cell wall homeostasis [43]. Therefore, Ca^2+^ is hypothesized to play double-edged sword” role in the granulation process, and the disruption of its concentration balance is an important cytological basis for quality deterioration.

## 5. Conclusions

Taking water-osmotic physiology as the guiding framework and based on time-series measurements over 200 d of storage and grade-specific expression profiling, we characterized the sequence of physiological and molecular events associated with postharvest granulation in ‘Newhall’ navel oranges. Granulation onset was closely associated with a preferential decline in peel water potential, which reached its minimum (−2.96 MPa) at 80 d and coincided with a peak peel–pulp water potential gradient of 1.85 MPa; because the present dataset documents a temporal association rather than a direct causal test, this gradient is best regarded as a plausible physical driver of water migration from the juice sacs toward the peel. Water loss occurred together with the aberrant activation of cell wall metabolism, which continuously consumes osmoregulatory solutes such as sugars and acids and appears to form a positive feedback loop with the collapse of osmotic adjustment. In parallel, the K, Ca, and Mg concentrations measured in the two tissues are consistent with a redistribution of these elements from the peel toward the pulp along the water potential gradient following ion leakage from impaired membranes; because this migration was inferred from tissue concentrations rather than measured directly, it is presented here as a well-supported hypothesis rather than an established fact. Among these elements, Ca^2+^ is proposed to be of particular relevance to the physical symptoms of granulation: released Ca^2+^ re-cross-links with demethylesterified pectin to form “egg-box” gel structures, which trap free water within the cell wall and, by limiting the access of polygalacturonases to the pectin backbone, restrict pectin degradation and contribute to cell wall stiffening; the resulting rigid, densely cross-linked matrix cannot undergo the reversible stretching that underlies turgor-driven elasticity, so that the juice sacs lose elasticity and become progressively firm and hardened, the characteristic textural change of granulation. We emphasize that this Ca^2+^–pectin egg-box mechanism is drawn from previous literature and is not directly demonstrated by the current study, as we did not quantify pectin esterification status or localize Ca^2+^ at the cell wall level. At the molecular level, the reprogrammed expression of tonoplast transporters is consistent with a disruption of vacuolar storage function that may further aggravate the imbalance between water and solute homeostasis: *CsTIP* members were strongly upregulated in the pulp, which would be expected to favour vacuolar water release, and the sugar transporters *NPF5.1*, *NPF8.3*, *SUT4*, *STP7*, *TMT2*, *EDR6L5*, and *EDR6L6* (1.88–3.36-fold) together with *ABGC15* (2.81–5.56-fold) were upregulated, whereas the organic acid transporters *ALMT4*/*ABCC4* were downregulated. The upregulation of *SUT4* and related sugar transporters is of particular significance for the osmotic imbalance observed, because these proteins export sucrose and other sugars out of the vacuole, thereby depleting the solute pool that lowers vacuolar osmotic potential and reducing the capacity of the juice sacs to retain water. Correlation analysis further showed that granulation severity was closely related to water potential, water content, and tonoplast transporter expression. Taken together, these findings suggest that postharvest granulation is a cascade process progressing from water potential gradient formation and osmotic adjustment collapse to molecular dysregulation of tonoplast transport. This model remains a working hypothesis, and provides a theoretical basis for delaying granulation by regulating fruit water dynamics and tonoplast transport activity.

## Data Availability

The original contributions presented in this study are included in the article/Supplementary Material. Further inquiries can be directed to the corresponding author.

## Supplementary Materials

The following supporting information can be downloaded at https://www.mdpi.com/article/10.3390/foods15213848/s1, Figure S1: Changes in fruit accumulated water loss (A), average water loss (B) of navel orange during ambient storage; Table S1: Sequences of qRT-PCR primers for *CsTIPs*, tonoplast-localized sugar and acid transporter genes.
