# Supplementary material for: Water Physiological Characteristics and Tonoplast Transporter Expression Profiles During Granulation in ‘Newhall’ Navel Oranges
Source: Foods. 2026 Oct 28;15(21):3848. doi: 10.3390/foods15213848 (PMC13763533; doi:10.3390/foods15213848)
Supplement: Supplementary file 1 [file foods-15-03848-s001.zip › Supplemental Figure S1.pdf]

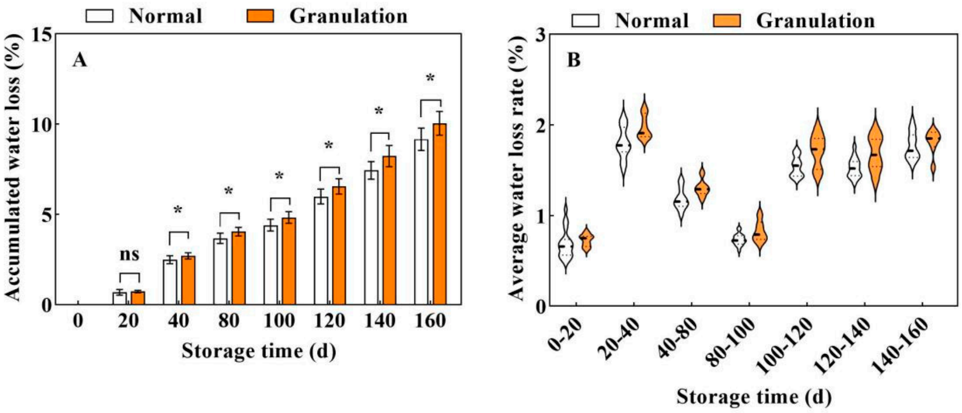

Figure S1. Changes in fruit accumulated water loss (A), average water loss (B) of navel orange during ambient storage.

Note: Data are presented as mean ± SD, with n ≥ 3. ns, not significant; *, $P < 0.05$.
